# Exploration and validation of a combined Hypoxia and m6A/m5C/m1A regulated gene signature for prognosis prediction of liver cancer

**DOI:** 10.1186/s12864-023-09876-3

**Published:** 2023-12-14

**Authors:** Min ren, Bei Fan, Guangcai Cao, Rongrong Zong, Liaoliao Feng, Huiru Sun

**Affiliations:** 1 College of Life Science, Yan’an University, 716000, Yan’an, China; 2 The First Clinical Medical College, Yan’an University, 716000, Yan’an, China

**Keywords:** Liver cancer, Hypoxia, m6A, m5C, m1A RNA methylation, Prognosis

## Abstract

**Background:**

It is widely acknowledged that hypoxia and m6A/m5C/m1A RNA modifications promote the occurrence and development of tumors by regulating the tumor microenvironment. This study aimed to establish a novel liver cancer risk signature based on hypoxia and m6A/m5C/m1A modifications.

**Methods:**

We collected data from The Cancer Genome Atlas (TCGA-LIHC), the National Omics Data Encyclopedia (NODE-HCC), the International Cancer Genome Consortium (ICGC), and the Gene Expression Omnibus (GEO) databases for our study (GSE59729, GSE41666). Using Cox regression and least absolute shrinkage and selection operator (LASSO) method, we developed a risk signature for liver cancer based on differentially expressed genes related to hypoxia and genes regulated by m6A/m5C/m1A modifications. We stratified patients into high- and low-risk groups and assessed differences between these groups in terms of gene mutations, copy number variations, pathway enrichment, stemness scores, immune infiltration, and predictive capabilities of the model for immunotherapy and chemotherapy efficacy.

**Results:**

Our analysis revealed a significantly correlated between hypoxia and methylation as well as m6A/m5C/m1A RNA methylation. The three-gene prognosis signature (*CEP55*, *DPH2*, *SMS*) combining hypoxia and m6A/m5C/m1A regulated genes exhibited strong predictive performance in TCGA-LIHC, NODE-HCC, and ICGC-LIHC-JP cohorts. The low-risk group demonstrated a significantly better overall survival compared to the high-risk group (*p* < 0.0001 in TCGA, *p* = 0.0043 in NODE, *p* = 0.0015 in ICGC). The area under the curve (AUC) values for survival at 1, 2, and 3 years are all greater than 0.65 in the three cohorts. Univariate and Multivariate Cox regression analyses of the three datasets indicated that the signature could serve as an independent prognostic predictor (*p* < 0.001 in the three cohorts). The high-risk group exhibited more genome changes and higher homologous recombination deficiency scores and stemness scores. Analysis of immune infiltration and immune activation confirmed that the signature was associated with various immune microenvironment characteristics. Finally, patients in the high-risk group experienced a more favorable response to immunotherapy, and various common chemotherapy drugs.

**Conclusion:**

Our prognostic signature which integrates hypoxia and m6A/m5C/m1A-regulated genes, provides valuable insights for clinical prediction and treatment guidance for liver cancer patients.

**Supplementary Information:**

The online version contains supplementary material available at 10.1186/s12864-023-09876-3.

## Introduction

Liver cancer, the second most deadly malignancy worldwide, is characterized by a poor prognosis [1]. Existing diagnostic tools for liver cancer relying on histological and radiological assessments, often lack precision and practicality [2]. Moreover, clinicopathological features have failed to account for the heterogeneity, among patients, resulting in varying treatment outcomes even among individuals with the same TNM stage [3]. Current staging methods also fall short in guide immunotherapy, which has shown promise in treating liver cancer [4, 5]. Hence, there is pressing need for the development of a novel liver cancer signature capable of predicting prognosis and guiding treatment decisions.

The presence of hypoxia within tumor microenvironment (TME) has been attributed to the considerable distance between the vascular system and tumor cells, coupled with the rapid proliferation of tumor cells [6, 7]. As oxygen is becomes increasingly scarce, tumor tissues establish a new dynamic equilibrium, leading to the formation a hypoxia, hypoglycemic and acidic TME that favors tumor growth [8, 9]. Extensive research has established a strong correlation between hypoxia and rapid tumor progression, metastasis, and drug resistance [10–13]. Notably, liver cancer is one of the most hypoxic malignant tumors, with a median oxygen content as low as 0.8% [14]. The hypoxic TME induces metabolic reprogramming, mediated by mRNA methylation, which subsequently triggers phenotypic changes in immune cells, creating an immunosuppressive TME [15].

Eukaryotic mRNA undergoes methylation modifications including N^6^-methyladenosine (m6A), 5-methylcytosine (m5C), and N^1^-methyladenosine (m1A). Research has revealed that genes involved in the regulation of m6A/m5C/m1A modifications play a pivotal role in shaping the TME, thereby promoting tumor progression [16–19]. Hypoxia-inducible factors (HIFs) orchestrate cellular adaptation to hypoxic environments by participating in multiple regulatory pathways [20, 21]. HIFs activation governs glucose and lactate metabolism, enabling tumors to thrive in hypoxia conditions while also contributing to the formation of an immunosuppressive TME [22–25]. Studies have demonstrated that m6A-modifying enzymes influence the methylation and expression of HIFs, thus affecting tumor proliferation. For example, in glioblastoma, hypoxia-induced ALKBH5 stabilizes SFPQ on the CXCL8 gene by clearing m6A methylated NEAT1, promoting the expression of CXCL8/IL8 and facilitating immune evasion [26]. Upregulation of HBXIP can enhance the expression of m6A methylase METTL3, maintaining elevated levels of HIF-α and promoting the malignant proliferation of liver cancer [27]. Hypoxia within the TME alters the methylation levels of m6A/m5C/m1A, subsequently affecting downstream adaptive responses such as immune cell function and tumor behavior [28–30]. It has been reported that there is a positive feedback loop between hypoxia and m6A/m5C/m1A methylation in driving malignant tumor proliferation [15].

Therefore, in the present study, we analyzed the interplay between hypoxia and m6A/m5C/m1A regulated genes and established a novel risk prognosis signature for liver cancer that integrated both hypoxia and m6A/m5C/m1A modification. To our knowledge, this represents the first prognostic signature that combines hypoxia and mRNA methylation regulatory genes, offering an enriching approach to clinical management and providing valuable guidance for the neoadjuvant treatment of liver cancer.

## Materials and methods

### Dataset collection and preprocessing

The flow chart outlining our research is illustrated in Fig. S1. We collected RNA-seq, methylation-seq, and clinicopathological data for liver cancer samples from various sources. Specifically, TCGA-LIHC and ICGC-JP were acquired from Xena (https://xenabrowser.net/datapages/), NODE-OEP000321 were downloaded from NODE (https://www.biosino.org/node/), and GSE59729, GSE41666, and GSE144269 were obtained from GEO (https://www.ncbi.nlm.nih.gov/gds). The clinical information of these data sets is listed in Table 1. To facilitate analysis, we converted all transcriptome sequencing data from count to TPM format and retrieved chip data using R package GEOquery (version 2.64.2) [31]. Subsequently, we standardized all gene expression profiles using the normalizeBetweenArrays function from the R package limma (only for chip data) and log2(X + 1) transformation [32]. The TCGA-LIHC dataset was employed as the training set for model development, while NODE-OEP000321 and ICGC-JP served as the validation sets. All analyses were performed using R (version 4.2.2). GSE144269, a transcriptome sequencing dataset for liver cancer, was used to verify the expression levels of risk genes.

**Table 1:** Summary of clinical information from the datasets used in the analysis

	Training cohort	Validation cohorts	
	TCGA (362)	NODE (159)	ICGC (231)
status (%)			
alive	233 (64.4)	103 (64.8)	188 (81.4)
dead	129 (35.6)	56 (35.2)	43 (18.6)
time (mean (SD))	2.22	2.66 (1.11)	67.20 (10.12)
age (mean (SD))	59.52	53.69 (10.90)	2.22 (1.14)
stage (%)			
I&II	254 (70.2)	105 (66.0)	141 (61.0)
III&IV	84 (23.2)	54 (34.0)	90 (39.0)
NA	24 (6.6)	-	-
gender (%)			
female	118 (32.6)	31 (19.5)	61 (26.4)
male	244 (67.4)	128 (80.5)	170 (73.6)
T_stage (%)			
T1&T2	271 (74.9)	-	-
T3&T4	88 (24.3)	-	-
NA	3 (0.8)	-	-
bmi (mean (SD))	26.14 (8.47)	-	-
tumor_grade (%)			
G1	55 (15.2)	-	-
G2	174 (48.1)	-	-
G3	116 (32.0)	-	-
G4	12 (3.3)	-	-
NA	5 (1.4)	-	-

Hypoxia genes were extracted from the hallmark gene set in the Molecular Signatures Database (MsigDb, www.gsea-msigdb.org, version 7.0). The regulatory genes (Writer, Reader, and Eraser) of m6A/m5C/m1A were obtained from the literature [33, 34].

### Unsupervised clustering of hypoxia and its correlation with methylation

To assess the hypoxia status of liver cancer patients, we applied an unsupervised clustering algorithm by the R package ConsensusClusterPlus (version 1.60.0) (default parameter) based on the hypoxia hallmark gene set [35]. Additionally, we computed the activation scores for hypoxia and m6A/m5C/m1A regulation using the single sample Gene Set Enrichment Analysis (ssGSEA) method from the R package GSVA (version 1.44.2). We analyzed differences in the expression of m6A/m5C/m1A regulator genes and total methylation levels across different hypoxia clusters. Furthermore, we examined the correlation between hypoxia and the activation of m6A/m5C/m1A regulation [36].

### Established a Hypoxia-m6A/m5C/m1A-related-score scoring system to evaluate liver cancer cases

To identify differentially expressed genes (DEGs) associated with hypoxia status more accurately, we intersected DEGs obtained from hypoxia clustering (TCGA-LIHC) and differential oxygen treatment (GSE41666 and GSE59729). We then calculated DGEs using the R package limma (|logFoldChange|> 1, *p* < 0.01) [32]. Subsequently, we analyzed DEGs between tumor and normal samples using the same package (|logFoldChange|> 1, *p* < 0.01) and identified gene sets significantly related to m6A/m5C/m1A regulator genes (*r* > 0.5, *p* < 0.001). The intersection of these two gene sets yielded a candidate gene set for model establishment using joint Cox regression analysis. Initially, we employed the Univariate Cox regression to identify genes related to overall survival (OS) (selecting genes with p values less than 0.05 after adjusting for p values using the Benjamini & Hochberg correction) via the R package Survival (version 3.5–5). Subsequently, we employed the Least Absolute Shrinkage and Selection Operator (LASSO) penalized Cox regression model to mitigate overfitting among candidate genes using the R package glmnet (version 4.1–7) and Survival. Finally, a risk model was established using Multivariate Cox regression, and risk scores for each patient were computed as follows:

$$\mathrm{risk~score}~({\text{patient}})=\sum_{k=1}^{n} \left({\text{coef}} \, \times {\text{exp}}\right)$$

where n means all samples, exp indicates the expression level for each risk gene, and coef indicates their regression coefficients.

### Evaluation and verification of the prognostic signature

We assessed the predictive and generalization capabilities of the Hypoxia-m6A/m5C/m1A-related-Score (HMRs) gene signature in both the training and validation sets. Patients were stratified into high- and low-risk groups based on the median risk score. We analyzed survival differences between these groups using Kaplan–Meier (K-M) curves with the R package survminer (version 0.4.9) and evaluated the predictive capability of the risk score through Received Operating characteristic curve (ROC) analysis. Univariate and Multivariate Cox regression analyses were conducted to assess the clinical prognostic independence of the prognostic risk model. Principal Component Analysis (PCA) and t-Distributed Stochastic Neighbor Embedding (t-SNE) were employed to evaluate the classification ability of the HMRs signature.

### Relationships between HMRs gene signature and genomic alterations

It is well-established that cancer patients with different prognoses can distinguish variations in gene expression and mutation patterns. Consequently, we investigated the association between risk groups determined by HMRs and genomic alterations. Initially, we performed an analysis of DEGs between high- and low-risk groups using the R package limma (|logFoldChange|> 0.5, *p* < 0.05). To gain insights into the functional relevance of these DEGs, we conducted pathway enrichment analysis using Metascape [37]. Subsequently, we utilized the R package progeny (version 1.18.0) to assess the activation levels of 14 typical cancer related pathways between the high- and low-risk groups. We then explored the relationship between genomic instability and risk scores in liver cancer from various angles. This included an analysis of the mutation spectrum and somatic total mutation burden (TMB) using the R package maftools (version 2.12.0) and an examination of copy number variation (CNV) using GISTIC2 [38, 39]. Additionally, we investigated differences in fragmented genomic alterations and Homologous Recombination Deficiency (HRD) between the two risk groups [40, 41]. Cancer stem cells, which exhibit characteristics akin to normal stem cells, play a pivotal role in drug resistance, proliferation, and metastasis of tumors [42]. To better understand their involvement, we compared tumor stem cell indices between the high- and low-risk groups [43].

### Relationships between HMRs gene signature and immunocyte infiltration

It is widely acknowledged that the immune microenvironment of tumor tissue has a profound impact on tumor progression and prognosis [44]. Therefore, we examined the relationship between the immune microenvironment of liver cancer and the HMRs gene signature from various angles. Initially, we utilized the R package estimate (version 1.0.13) to compute the immune score of the tumor [45]. Subsequently, we employed the ssGSEA method from the R package GSVA (version 1.44.2) to estimate the proportions of 28 typical immune cell types [36]. To comprehensively assess the immune microenvironment of liver cancer, we employed a variety of immunoinfiltration analysis methods, including TIMER, CIBERSORT, XCELL, QUANTISEQ, MCP-counter, EPIC, and CIBERSORT-ABS. All proportions of immune cells were retrieved from TIMER2.0 (http://timer.comp-genomics.org/) [46–52]. To evaluate associations between immune cells and risk scores, we employed Wilcoxon signed-rank sum tests and Pearson correlation analyses. Finally, we examined the activation of the immune cycle in liver cancer based on the Tracking Tumor Immunophenotype (TIP) tool (http://biocc.hrbmu.edu.cn/TIP/index.jsp) [53].

### Estimate of immunotherapeutic and chemotherapy drug response between HMRs groups

To broaden the model’s applicability, we analyzed its potential impact on clinical practice. Initially, we assessed the predictive power of risk scores for immunotherapy outcomes. We conducted this analysis using the PD-1 treatment cohort from IMvigor210 to explore the relationship between HMRs and immunotherapy response [54]. Additionally, we then evaluated the predictive effect of HMRs on immunotherapy using Tumor Immune Dysfunction and Exclusion (TIDE, http://tide.dfci.harvard.edu/faq/), tumor inflammation score (TIS), and immunophenoscore (IPS, obtained from The Cancer Immunome Atlas Database, https://tcia.at/about) [55–58]. Furthermore, we compared differences in common immune checkpoint inhibitors (ICI) and the Human Leukocyte Antigen (HLA) family genes among the HMRs groups. Transcatheter arterial chemoembolization (TACE) serve as a primary treatment for unresectable liver cancer. However, its therapeutic efficacy varies due to the heterogeneity of liver cancer. We analyzed the ability of the risk score to predict TACE response based on GSE104580. Finally, we utilized the Genomic of Drug Sensitivity in Cancer (GDSC) and the R package pRRophetic (version 0.5) to predict the half-maximal inhibitory concentration (IC50) of 8 common liver cancer chemotherapeutic drugs. We compared differences in IC50 values between the HMRs groups by using Wilcoxon signed-rank sum tests [59, 60].

### Multi-database validation of risk gene expression and function analysis of candidate gene

To validate the findings, we leveraged multiple independent datasets to analyze the differential expression of risk genes between tumor and normal tissues. Initially, we identified the differentially expressed risk genes in the TCGA-LIHC, ICGC-JP, and GSE144269 datasets. Furthermore, we employed the online tool, The University of Alabama at Birmingham CANcer Data Analysis Portal (UALCAN, https://ualcan.path.uab.edu/index.html), to assess differences in risk gene expression at the protein level [61]. Immunohistochemical results for risk genes were obtained from The Human Protein Atlas (THPA) database (https://www.proteinatlas.org/) [62]. Finally, we examined the expression differences of risk genes in paired and unpaired tumor datasets using the online tool TNMplot (https://tnmplot.com/analysis/) [63].

We also conducted functional analyses of risk genes, including pan-cancer survival and differential expression analysis, using Gene Expression Profiling Interactive Analysis (GEPIA2, http://gepia2.cancer-pku.cn/#index) [64]. Additionally, differences in the methylation levels of genes under different clinical classifications were analyzed using UALCAN. To visualize the methylation sites of risk genes, we employed the R package trackViewer (version 1.36.2) to visualize [65]. Finally, we performed single-cell expression analysis of risk genes, initially assessing the expression levels of risk genes across different cell types in multiple liver cancer single-cell datasets using the Tumor Immune Single-cell Hub 2 (TISCH2, http://tisch.comp-genomics.org/home/) [66]. Subsequently, we determined in which cell types the risk genes were significantly expressed using the PanglaoDB database (https://panglaodb.se/index.html) [67]. Finally, the relationship between the expression levels of risk genes at both protein and transcript levels and the cell cycle were explored using the THPA database [62].

## Results

### Hypoxia-based clustering of liver cancer was significantly correlated with m6A/m5C/m1A methylation regulation

**Fig. 1:**
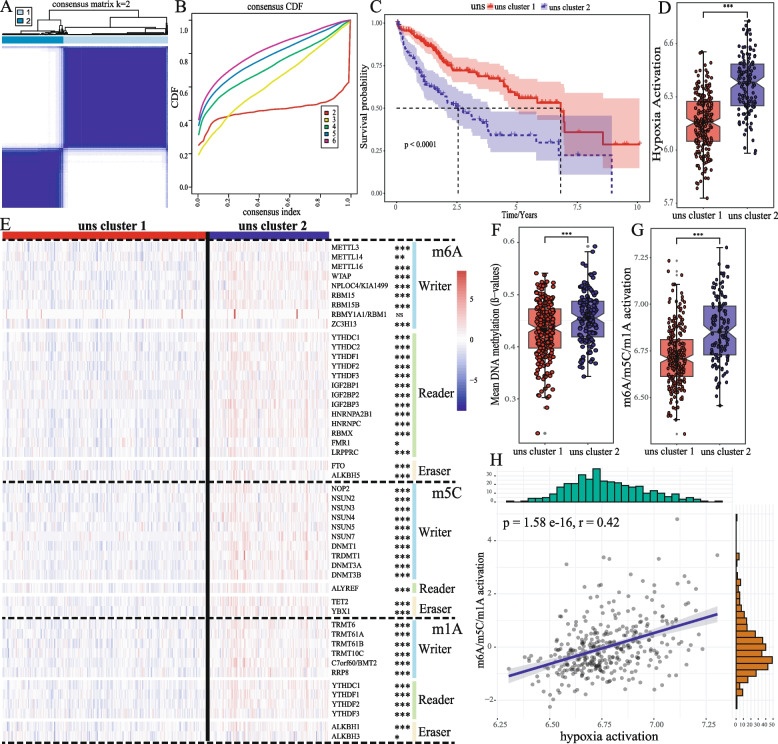
The relationship between hypoxia and m6A/m5C/m1A methylation regulation. **A** The consensus matrix of unsupervised clustering when K = 2. **B** Cumulative distribution function (CDF) curve proves that K = 2 has the best clustering effect. **C** K-M survival curves showed the differences of overall survival rate among the 2 clusters. **D** Analysis of activation difference of hypoxia pathway between two clusters, the significance of the difference was analyzed by Wilcoxon signed rank test. **E** Heatmap of differential expression of m6A/m5C/m1A methylation regulation genes in two clusters, the significance of the difference was analyzed by T test. **F** Analysis of differences in Mean DNA methylation degree between two clusters, the significance of the difference was analyzed by Wilcoxon signed rank test. **G** Analysis of activation differences of m6A/m5C/m1A methylation regulation between two clusters, the significance of the difference was analyzed by Wilcoxon signed rank test. **H** Correlation analysis between hypoxia pathway and m6A/m5C/m1A methylation regulation. (*p < 0.05, **p < 0.01, ***p < 0.001, ****p < 0.0001, NS means non-significant)

The results of unsupervised clustering showed that hypoxia gene sets could classify liver cancer into two clusters (Fig. 1A, B), with significant differences in survival between these two clusters (Fig. 1C). Pathway activation analysis based on ssGSEA showed that the activation degree of hypoxia in cluster 2 was significantly higher than that in cluster 1 (Fig. 1D). Almost all m6A/m5C/m1A regulator genes were significantly highly expressed in cluster 2 (Fig. 1E), and the average methylation degree in cluster 2 was significantly higher than that in cluster 1 (Fig. 1F). Pathway activation analysis based on ssGSEA showed that the activation degree of m6A/m5C/m1A in cluster 2 was significantly higher than in cluster 1 (Fig. 1G), and there was a significant correlation between hypoxia pathway activation of m6A/m5C/m1A regulation pathway (Fig. 1H, *r* = 0.42). These results suggest that hypoxia is positively correlation with m6A/m5C/m1A mediated methylation regulation.

### Construction of HMRs model of liver cancer

**Fig. 2:**
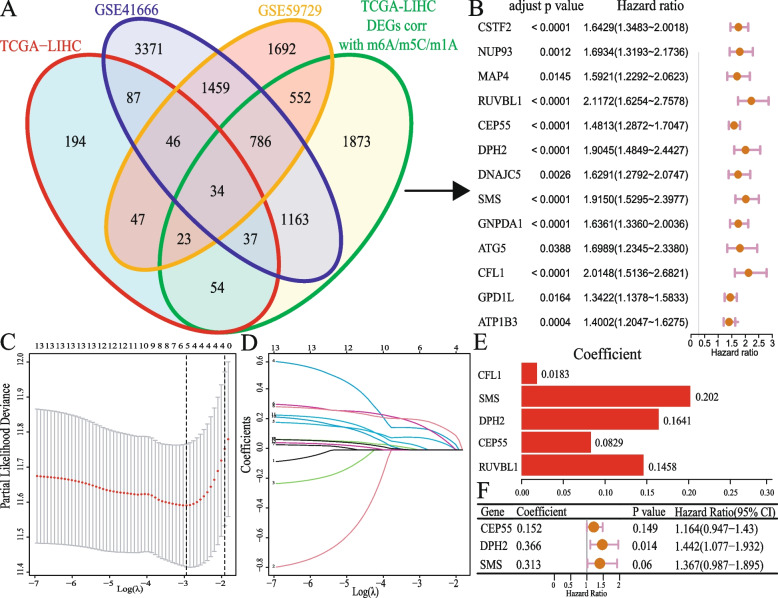
Construction of HMRs model of liver cancer **A** Venn diagram displaying the 34 intersection genes of the hypoxia-associated DEGs and the m6A/m5C/m1A methylation regulation associated DEGs. **B** Forest plots shows the 13 survival related genes obtained by Univariate Cox regression; P value corrected using B-H correction. **C** LASSO deviance profile of the 13 genes. **D** LASSO regression coefficient profile of the 13 genes. **E** 5 genes coefficient after LASSO analysis. **F** Final 3 genes forest plot after Multivariate Cox regression

A total of 34 genes related to hypoxia, m6A/m5C/m1A methylation regulation, and differentially expressed in tumors were used as candidate genes for model creation (Fig. 2A). 13 genes related to prognosis were obtained through Univariate Cox regression analysis (Fig. 2B), and candidate genes were screened using LASSO Cox regression (Fig. 2C, D, E) and Multivariate Cox regression (Fig. 2F). Finally, a liver cancer risk score model related to hypoxia and m6A/m5C/m1A methylation regulation was obtained. The risk score was estimated as follows:

$$\mathrm{Risk~score }= (0.152 \times \mathrm{ expr~of~CEP}55) + (0.366 \times \mathrm{expr~of~DPH}2) + (0.313 \times \mathrm{expr~of~SMS})$$

### Validation of the prognostic prediction efficacy of HMRs model in liver cancer

**Fig. 3:**
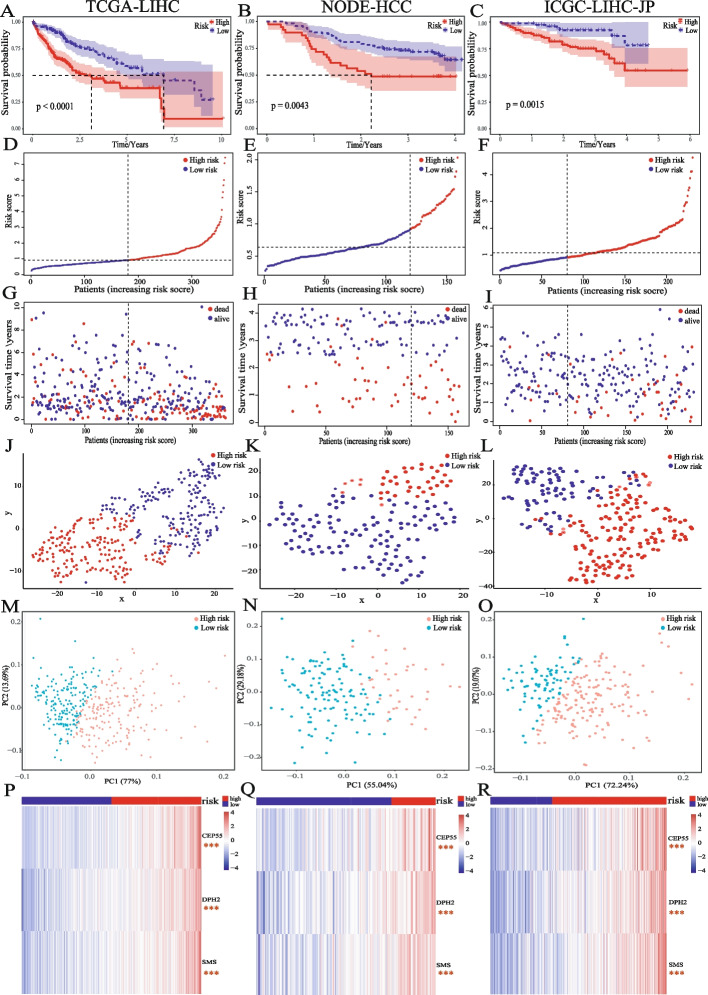
Validation of the prognostic prediction efficacy of HMRs model **A B C** K-M curve of OS of liver cancer patients in high- and low-risk groups. **D E F** Risk curve for the investigated patients. **G H I** Risk scatter diagram for the investigated patients. The investigated patients risk scores clustering based on t-SNE **J K L** and PCA **M** **N O** **P Q R** Heatmap for the different expression of 3 risk genes in high- and low-risk group, the significance of the difference was analyzed by T test, the red asterisk represents significantly higher expression in the latter group. (*p < 0.05, **p < 0.01, ***p < 0.001, ****p < 0.0001, NS means non-significant)

Based on the HMRs model, we calculated the risk scores of patients in each dataset and divided all patients into high-risk and low-risk groups based on the median risk scores in the training set. Survival analysis results showed that patients in the high-risk group had a worse prognosis (Fig. 3A, B, C, *p* < 0.01) and higher mortality (Fig. 3D-I). t-SNE and PCA analysis showed significant clustering of patients in the high- and low-risk groups (Fig. 3J, K, L for t-SNE, Fig. 3M, N, O for PCA). Risk genes were significantly high expression in the high-risk group (Fig. 3P, Q, R). These results indicated that the HMRs model is a robust prediction classifier for liver cancer prognosis.

**Fig. 4:**
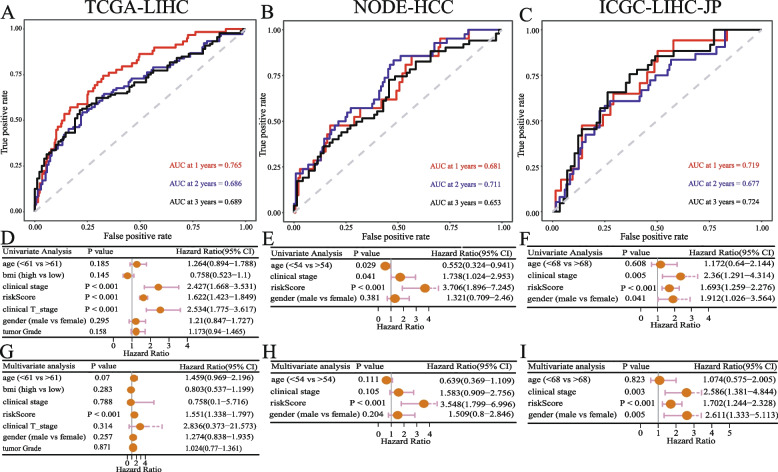
Validation of the prognostic independence of the HMRs model **A B C** The 1-, 2-, 3-year AUC in ROC analysis. **D E F** Univariate and **G H I** Multivariate Cox regression analysis of risk score and clinical features

The time-dependent ROC curve revealed that the HMRs model had good predictive performance in both the training and validation sets. The area under the curve (AUC) of 1-year, 2-year, and 3-year of the training set and validation set were greater than 0.65 (0.765, 0.686, 0.689 in TCGA-LIHC, 0.681, 0.711, 0.653 in NODE-HCC, 0.719, 0.677, 0.724 in ICGC-LIHC-JP) (Fig. 4A-C). The results of prognostic independence analysis based on Univariate and Multivariate Cox regression indicated that the HMRs model was an independent prognostic factor for liver cancer (Fig. 4D-I). Univariate Cox regression analysis identified clinical stage and risk score as risk factors for liver cancer (*p* < 0.001 in all three data sets). Multivariate Cox regression analysis showed that risk score was the only independent prognostic factor in TCGA-LIHC and NODE-HCC datasets. Analysis of the ICGC-LIHC-JP dataset revealed clinical stage, risk score, and gender as risk factors for liver cancer (*p* < 0.05). These results strongly suggest that HMRs represent an independent predictor of the prognosis of liver cancer patients.

### The HMRs model is significantly correlated with genomic alterations

Due to the significant correlation between the HMRs model and the prognosis of liver cancer patients, we conducted a series of bioinformatics analyses to reveal the molecular mechanisms associated with this model.

**Fig. 5:**
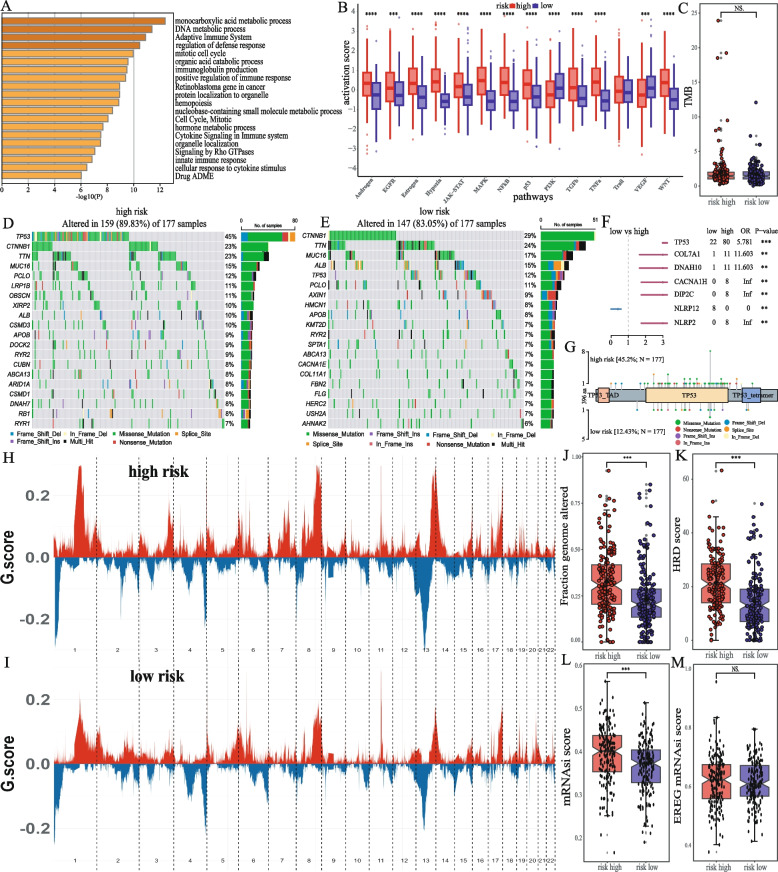
Relationship between risk models and genomic alterations **A** Pathway annotation of DEGs between risk groups. **B** Analysis of differences in activation of 14 typical cancer-related pathways in the high- and low-risk group, the significance of the difference was analyzed by Wilcoxon signed rank test. **C** Analysis of differences in TMB between two risk groups, the significance of the difference was analyzed by Wilcoxon signed rank test. **D E** The mutation landscape of patients in the TCGA-LIHC cohort (show the top 20 genes) about the high- and low-risk groups. **F** Display of mutation genes information with significant differences between risk groups. **G** Display of mutation sites of TP53 differences between high- and low-risk groups. Display CNV information of high **H** and low **I** risk groups. **J** Analysis of differences in fraction genome altered between two risk groups, the significance of the difference was analyzed by Wilcoxon signed rank test. **K** Analysis of differences in HRD score between two risk groups, the significance of the difference was analyzed by Wilcoxon signed rank test. Analysis of tumor cell stemness index based on methylation **L** and epigenetic regulation characteristics **M** between two risk groups, the significance of the difference was analyzed by Wilcoxon signed rank test. (*p < 0.05, **p < 0.01, ***p < 0.001, ****p < 0.0001, NS means non-significant)

A total of 893 genes (479 down-regulated, 414 up-regulated) were differentially expressed between high- and low-risk groups (Fig. S2). Pathway analysis showed that DEGs were significantly enriched in processes such as metabolism (monocarboxylic acid metabolic process, DNA metabolic process) and immune response (adaptive immune system) (Fig. 5A). Activation analysis of typical tumor pathways showed that pathways related to cancer progression, such as Androgen, EGFR, Estrogen, Hypoxia, JAK-STAT, and MARK were significantly activated in the high-risk group (Fig. 5B). However, pathways related to cancer suppression and immune response, such as NFKB, p53, TGFβ, TNFα, and WNT, were also significantly activated in the high-risk group (Fig. 5B). Pathways related to cell proliferation and migration, such as PI3K and VEGF, were significantly activated in the low-risk group (Fig. 5B). These results indicate a correlation between the HMRs model and pathways such as cell proliferation and immune regulation.

We then analyzed the differences in gene mutations between high- and low-risk groups. Although there was no significant difference in TMB between the two groups (Fig. 5C), there were differences in mutation landscape between the high- and low-risk groups (Fig. 5D, E). Genes such as TP53, COL7A1, DNAH10, CACNA1H, DIP2C, and NLRP2 exhibited a higher mutation frequency in the high-risk group, while NLRP12 has a higher mutation frequency in the low-risk group (Fig. 5F). Mutation site analysis showed that the type and number of mutations in TP53 were significantly higher than those in the low-risk group (Fig. 5G). These results suggest the presence of differences in gene mutation patterns among patients in the high- and low-risk groups, and there is significant heterogeneity between the two groups of patients. Genomic variation analysis showed that the high-risk group had more CNV and structural variations (Fig. 5H, I, J). The higher HRD score in the high-risk group indicated that there were more defects in the DNA damage repair mechanism in the high-risk group, leading to more mutations (Fig. 5K). The cancer stem cell index based on methylation suggested that the stemness characteristics of the high-risk group were more obvious (Fig. 5L), while the results based on the epigenetic regulation characteristics of stem cells suggested that there was no significant difference between the two groups (Fig. 5M). These results indicate significant differences in expression patterns and gene mutations between high- and low-risk groups, leading to survival differences between groups.

### Analysis of immune microenvironment between risk groups

**Fig. 6:**
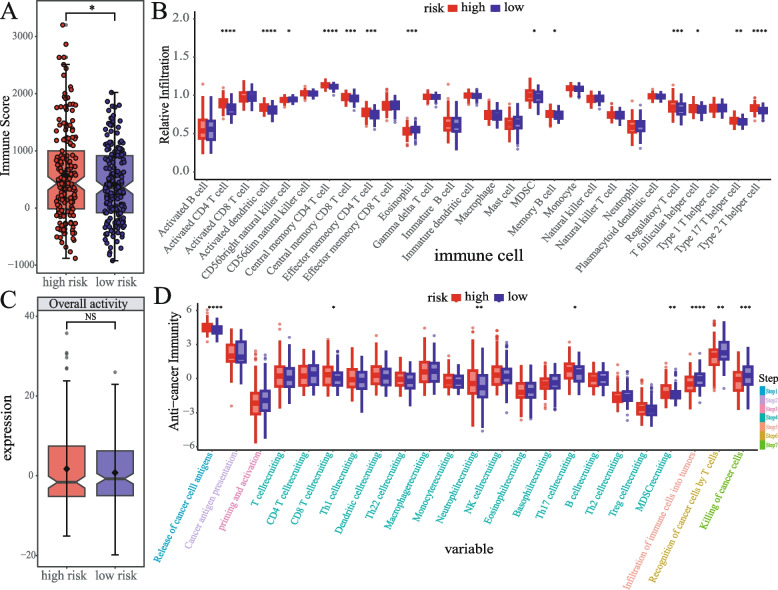
Analysis of tumor immune microenvironment **A** Analysis of differences in immune scores between two risk groups **B** Analysis of immune cell infiltration differences between two groups. **C** Overall immune activation differences between two risk groups based on TIP. **D** Differences in the cancer immunity cycle activities between two groups. The significance of the difference was analyzed by Wilcoxon signed rank test. (*p < 0.05, **p < 0.01, ***p < 0.001, ****p < 0.0001, NS means non-significant)

Immune infiltration analysis using the estimate suggested a higher degree of immune infiltration in the high-risk group (Fig. 6A), while there were no significant differences in tumor cell purity, stromal cell score, and estimate score (Fig. S3A, B, C). Immune cell infiltration analyses of 28 types of immune cells showed that Activated CD4 T cells, Activated dendritic cells, Central memory CD4 T cells, Central memory CD8 T cells, Effector memory CD4 T cells, MDSC, Memory B cells, Regulatory T cells, T follicular helper cells, Type 17 T helper cells, Type 2 T helper cells, were significantly activated in the high-risk group, while Eosinophils and CD56dim natural killer cells were highly infiltrated in the low-risk group (Fig. 6B). Other immune infiltration analysis approaches consistently suggested that the high-risk group has more abundant immune cell infiltration (Fig. S4A, B). The infiltrating immune cells in the high-risk group consisted of multiple types of immunosuppressive and activated cells, indicating a more complex immune microenvironment in the high-risk group. TIP analysis suggested that there was no significant difference in immune activation between high- and low-risk groups (Fig. 6C). Analysis of anti-tumor immune pathway activation showed that the high-risk group elevated activation levels in pathway associated with the Release of cancer cell antigens, CD8 T cell recruiting, Neutrophil recruiting, TH17 cell recruiting, and MDSC recruiting pathways in steps 1 and 4, while the Infiltration of immune cells into tumors, Recognition of cancer cells by T cells, Killing of cancer cells in steps 5, 6, 7 were significantly activated in the low-risk group (Fig. 6D). Taken together, these results indicated that the low-risk group could more effectively eliminate tumor cells, thereby improving the prognosis of patients.

### Prediction of treatment outcomes for liver cancer using the HMRs model

**Fig. 7:**
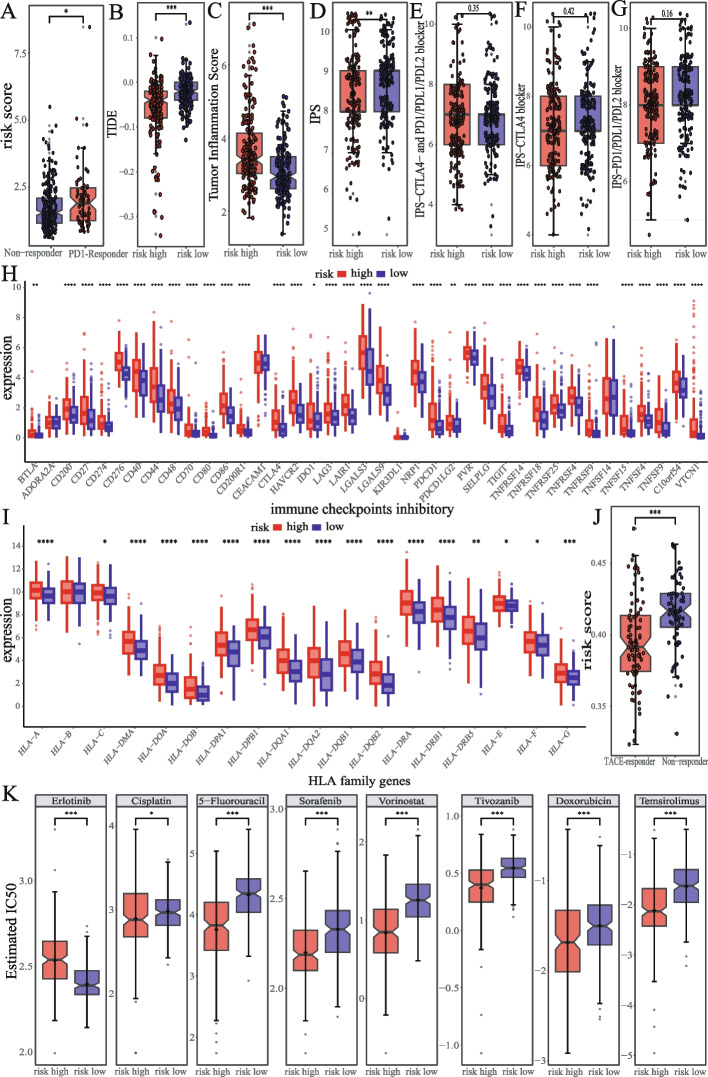
The ability of HMRs model to predict the outcome of liver cancer treatment **A** Association between risk scores and immunotherapy response based on the IMvigor210. **B** Difference in response to immunotherapy in risk groups predicted by TIDE. Differences in the TIS **C** IPS **D** IPS-CTLA4- and PD1/PDL1/PDL2 blocker **E** IPS-CTLA4 blocker **F**, IPS-PD1/PDL1/PDL2 blocker **G** ICIs **H** and HLA **I** between two groups. **J** Association between risk scores and TACE treatment response. **K** Difference in IC50 of common chemotherapy drugs between risk group. The significance of the difference was analyzed by Wilcoxon signed rank test. (*p < 0.05, **p < 0.01, ***p < 0.001, ****p < 0.0001, NS means non-significant)

Analysis of the IMvigor210-based immunotherapy dataset showed that patients who responded to treatment had higher risk scores (Fig. 7A). Evaluation of immunotherapy effectiveness based on TIDE suggested that the high-risk group had a lower TIDE score, indicating a better response to immunotherapy (Fig. 7B). TIS is considered a predictive biomarker for the combination therapy of PD-1 inhibitors, and patients with high TIS scores can benefit from treatment [56, 57]. Analysis showed that the TIS of the high-risk group was higher, indicating that immunotherapy combination therapy conferred more substantial benefits for the high-risk group (Fig. 7C). IPS analysis showed that the high-risk group had significantly higher IPS scores (Fig. 7D), while the remaining scores were comparable to the low-risk group (Fig. 7E, F, G). These results indicate that the high-risk group was more likely to benefit from immunotherapy. Multiple common ICIs and HLA were significantly expressed in the high-risk group (Fig. 7H, I), which also supports the speculation that the high-risk group can derive more benefit from immunotherapy. We also analyzed the predictive ability of our risk score for the effectiveness of TACE treatment. The results showed that TACE had a better effect on the low-risk group (Fig. 7J). Finally, we analyzed the guidance of risk scoring on chemotherapy medication. Of these 8 commonly used chemotherapy drugs, Erlotinib was more suitable for the low-risk group, while Cisplatin, 5-Fluorouracil, Vorinostat, Tivozanib, Doxorubicin, and Temsirolimus were more beneficial to the high-risk group (Fig. 7K).

### Analysis of expression patterns of risk genes

Due to the lack of clinical samples for risk gene validation in this study, we analyzed the expression patterns of risk genes from multiple perspectives. Analysis based on the ICGC-JP, GSE144269, and TCGA-LIHC datasets showed significant overexpression of risk genes in tumor tissues (Fig. S5A, B, C). Analysis based on the UALCAN database showed that the proteins of DPH2 and SMS were significantly overexpressed in tumors, while the difference in CEP55 was not significant (Fig. S5D). The results of immunohistochemistry analysis showed that CEP55 was moderately expressed in tumor tissue, with a lower expression level in normal tissue (Fig. S5E), while SMS exhibited low expression in tumor tissue and could not be detected in normal tissue (Fig. S5G). DPH2 was highly expressed in tumor tissues, while its expression level was low in normal tissues (Fig. S5F). We validated the expression patterns of risk genes using the expression chip data collected by TNM plot for liver cancer. The results showed that risk genes were significantly overexpressed in tumor tissues, both in paired and non-paired samples (Fig. S5H, I, J). In addition, pan-cancer analysis based on GEPIA2 showed that high expression of risk genes was associated with poor prognosis in various cancers (Fig. S6A, B, C, D, including liver cancer). Based on the above results, risk genes are significantly overexpressed in liver cancer’s tumor tissue and are all risk factors for liver cancer.

### Gene function analysis of DPH2

The results of multivariate Cox regression analysis highlighted the significant impact of *DPH2* on the prognosis of liver cancer (*p* = 0.014, hazard ratio > 1, Fig. 2F). We also confirmed that DPH2 was significantly overexpressed in both gene and protein expression levels in liver tumor tissue. Therefore, we analyzed the role of this gene in the development of liver cancer. Univariate and Multivariate Cox regression analysis showed that DPH2 was a significant independent prognostic factor in both the TCGA-LIHC (Fig. S7A, D) and ICGC-LIHC-JP (Fig. S7C, F) datasets, but nor significant in the NODE-HCC dataset (Fig. S7B, E). The time-dependent ROC curve analysis of DPH2 expression suggested that the AUC values of both TCGA-LIHC (Fig. S7G) and ICGC-LIHC-JP (Fig. S7I) datasets for survival at 1, 2, and 3 years are greater than 0.65, indicating that the gene has good predictive ability for liver cancer prognosis, while the NODE-HCC dataset revealed that DPH2 lacks predictive ability (Fig. S7H). Analysis shows that normal and low-level tumor-grade samples have higher levels of DPH2 methylation and lower gene expression (Fig. S7J, L). Patients with low gene expression levels were associated with higher methylation levels (Fig. S7K). Multiple methylation modification sites were observed in the promoter region of the DPH2 gene (Fig. S7M). These results suggest that DPH2 is a potential prognostic target in liver cancer, and methylation in the promoter region of this gene may affect its expression, thereby influencing clinical outcomes.

Further analysis showed that the correlation between DPH2 and immune cells is not significant (Fig. S6E). Analysis of liver cancer single-cell data based on TISCH2 showed that DPH2 exhibits low expression in immune-related cells (Fig. S8A). PanglaoDB database analysis showed that DPH2 is highly expressed in Basal cells (Fig. S8B). THPA database analysis demonstrated that the protein expression level of DPH2 increased with the progression of cell interphase, unlike gene expression (Fig. S8C, D, E, F). These results suggest a possible regulatory relationship between DPH2 and cell proliferation.

## Discussion

Liver cancer exhibits significant inter-tumor heterogeneous and involves complex and diverse biological processes, resulting in inconsistent prognoses [3, 68]. Given its high morbidity and mortality rates and limited treatment options, it is imperative to develop a convenient method for guiding personalized precision treatment for liver cancer.

Current evidence suggests that hypoxia within the TME is closely associated with unfavorable processes in liver cancer, including tumor proliferation, metastasis, angiogenesis, resistance to radio- and chemotherapy, and disease progression [69]. The impact of hypoxia on the TME is multifaceted, encompassing metabolic reprogramming and the modulation of immune cell functions through post-transcriptional methylation modifications [28]. Notably, there exists an intricate crosstalk between hypoxia in solid tumors and methylation modifications such as m6A/m5C/m1A [15]. In this study, we comprehensively examined the relationship between hypoxia and m6A/m5C/m1A methylation and subsequently developed a prognostic risk model for liver cancer by integrating hypoxia and methylation information. To our knowledge, this represents the first reported liver cancer prognosis model that integrates gene expression related to hypoxia and methylation regulation. Based on our comprehensive analyses, the HMRs model serves as a robust classifier for predicting liver cancer prognosis and offers valuable insights for guiding liver cancer immunotherapy and chemotherapy.

Our analysis preliminarily revealed a positive correlation between hypoxia and methylation in liver cancer, as patients with higher hypoxia pathway activation exhibited poorer prognoses, along with elevated methylation levels and increase m6A/m5C/m1A pathway activation. These finding validate the rationale behind our integrated modeling approach. Survival curves, clustering, ROC analysis, and both Univariate and Multivariate Cox analyses of the training and validation sets provided compelling evidence of the effectiveness and robustness of HMRs in predicting liver cancer prognosis.

Hypoxia has been linked to the promotion of tumor heterogeneity and genomic evolution, with high-hypoxia patients often displaying elevated CNV and TMB [70]. Additionally, hypoxia contributes to reshaping the TME by inducing m6A/m5C/m1A methylation, influencing biological functions such as immunosuppression, metabolic dysregulation, and metastasis promotion [15]. Studies have shown that genomic instability represented by TMB and abnormal immune microenvironment can reflect tumor prognosis and immunotherapy effect [71, 72]. In our study, we conducted a series of analyses using the TCGA-LIHC dataset to investigate the biological processes associated with HMRs. Our analyses revealed significant disparities in immune responses, cell proliferation, and metabolism pathways between high- and low-risk groups. Mutation of TP53 will affect cell apoptosis and cause the tumor to fall into a dynamic cycle of hypoxia [73]. Although no significant differences in TMB were observed between risk groups, the mutated genes were notably different, with TP53 mutations being more prevalent in the high-risk group, which also exhibited a higher frequency of CNV. Furthermore, we observed increased HRD in the high-risk group, suggesting greater genomic instability. Notably, hypoxia’s impact on gene expression, especially that of m6A/m5C/m1A regulators, has been shown to enrich and maintain tumor stem cells, consistent with our observation of a higher cancer stem cell index in the high-risk group [74, 75]. These finding collectively indicate that the HMRs model encompasses the characteristics of hypoxia and m6A/m5C/m1A methylation in regulating cancer biological processes, making it a potential prognostic predictor for liver cancer.

Further analysis revealed that the low-risk group exhibit a higher proportion of Eosinophils and CD56dim natural killer cells, potentially contributing to cancer cell suppression and improved survival. Eosinophils are thought to enhance immune function, while CD56dim natural killer cells inhibit cancer cell proliferation [76, 77]. Moreover, our assessment of anti-tumor immune pathway activation using the TIP tool suggested that the low-risk group may eliminate tumor cells more efficiently, contributing to better patient prognoses. Conversely, the high-risk group display complex immune cell infiltration with both immune-activating and immunosuppressive cell types exhibiting higher proportions. While immune activating cells like Central memory CD4 T cells, Central memory CD8 T cells and Type 2 T helper cells were more abundant in the high-risk group [78–80]. Immunosuppressive or inflammation-promoting cells like Effector memory CD4 T cells, MDSC and Regulatory T cells were also highly prevalent in the high-risk group [81, 82]. The effect of immune cells in TME on the occurrence and metastasis of hepatocellular carcinoma is complex [83]. This intricate immune cell composition in the high-risk group may renders anti-tumor pathways less effective, resulting in poorer prognoses.

Our results also indicated that the HMRs model could effectively assess the efficacy of immunotherapy and TACE for liver cancer. Despite limited immunotherapy datasets for liver cancer, multiple analysis methods consistently demonstrated that the high-risk group display better responses to immunotherapy. The high group’s increased release of cancer cell antigens and higher expression of multiple ICIs and HLAs suggest a potential advantage in responding to immunotherapy, supported by the release of more tumor antigens. Studies have shown that combined radiotherapy, chemotherapeutic, and immunotherapy can improve the prognosis of patients with liver cancer [84]. Our study also identified specific chemotherapeutic drugs that showed varying responses between risk groups, offering potential clinical benefits, particularly when combined with immunotherapy. TACE, on the other hand, exhibited better responses in the low -risk group, thereby providing valuable treatment guidance for patients in this subgroup.

Furthermore, we investigated the gene DPH2, known to be involved in ribosomal protein biosynthesis and protein elongation accuracy [85]. Expression analyses across multiple datasets consistently indicated significant overexpression of DPH2 in liver cancer. We observed a positive correlation between DPH2 expression levels and tumor stage, coupled with a negative correlation between methylation levels and both DPH2 expression and clinical phenotypes. These findings suggest a close association between DPH2 and mitosis. In normal tissues, promoter hypermethylation keeps DPH2 expression low, while in cancer tissues, decreasing methylation levels with increasing tumor stage elevate DPH2 expression potentially influencing disease progression. We propose that DPH2 represents a potential target for liver cancer prognosis evaluation and treatment.

## Conclusion

In summary, our study unveiled the intricate relationship between hypoxia and m6A/m5C/m1A methylation regulation, leading to the development of the Hypoxia-m6A/m5C/m1A-related-Score model. This model captures the biological phenotypes of hypoxia and m6A/m5C/m1A methylation, such as genomic instability, metabolic abnormalities, and immune dysregulation, effectively predicting liver cancer survival outcomes. Moreover, our model offers valuable insight into guiding immunotherapy, TACE, and chemotherapy for liver cancer, thus facilitating precise treatment strategies for liver cancer patients.

### Supplementary Information

**Additional file 1.**

## Data Availability

The original contributions presented in the study are included in the article/Supplement Material.
