# Supplementary material for: Exploration and validation of a combined Hypoxia and m6A/m5C/m1A regulated gene signature for prognosis prediction of liver cancer
Source: BMC Genomics. 2023 Dec 14;24:776. doi: 10.1186/s12864-023-09876-3 (PMC10722758; doi:10.1186/s12864-023-09876-3)
Supplement: Supplementary file 1 — Additional file 1. [file 12864_2023_9876_MOESM1_ESM.pdf]

Exploration and validation of a combined Hypoxia and m6A/m5C/m1A regulated gene signature for prognosis prediction of Liver Cancer

Min ren[1], Bei Fan[1], Guangcai Cao[2], Rongrong Zong[1], Liaoliao Feng[1], Huiru Sun[1, *]

1 College of Life Science, Yan'an university, Yan'an 716000, China

2 The First Clinical Medical Colledge, Yan'an University, Yan'an 716000, China

* Correspondence: shr@yau.edu.cn (H.S.)

Supplementary figures

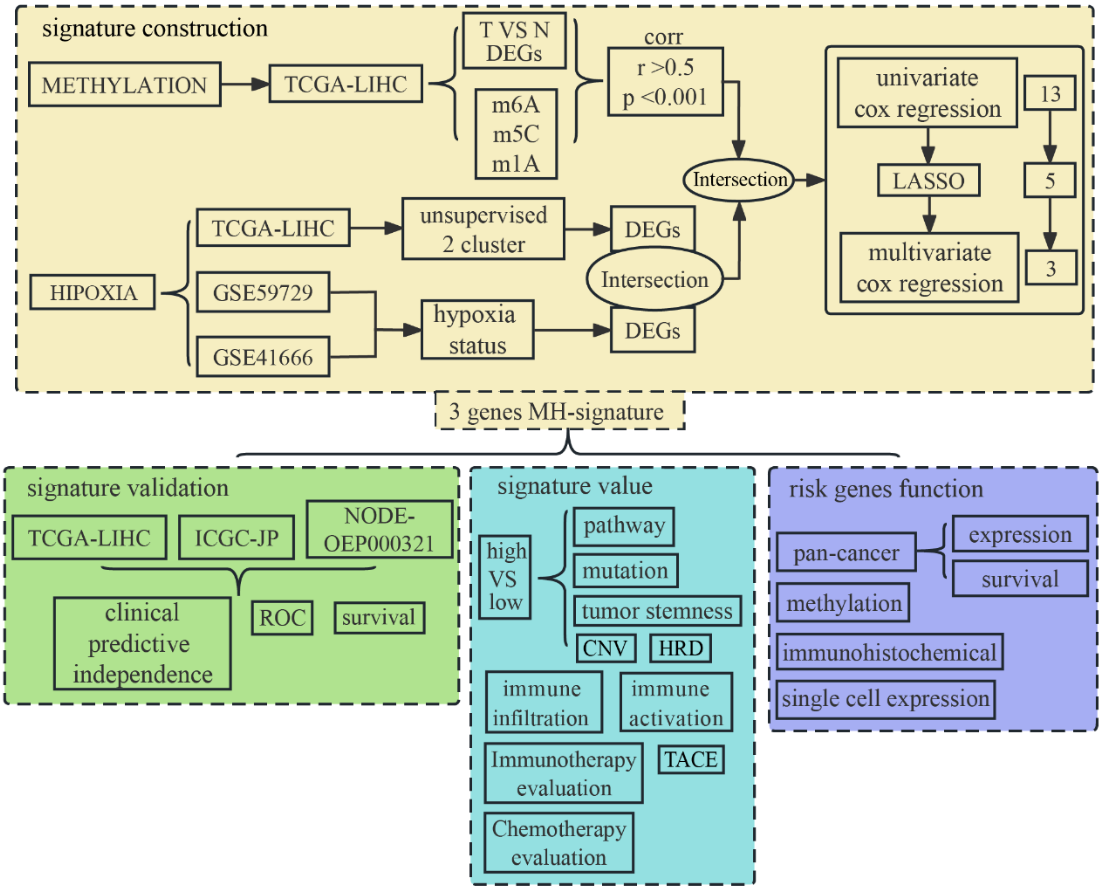

Figure S1 Flow chart of article.

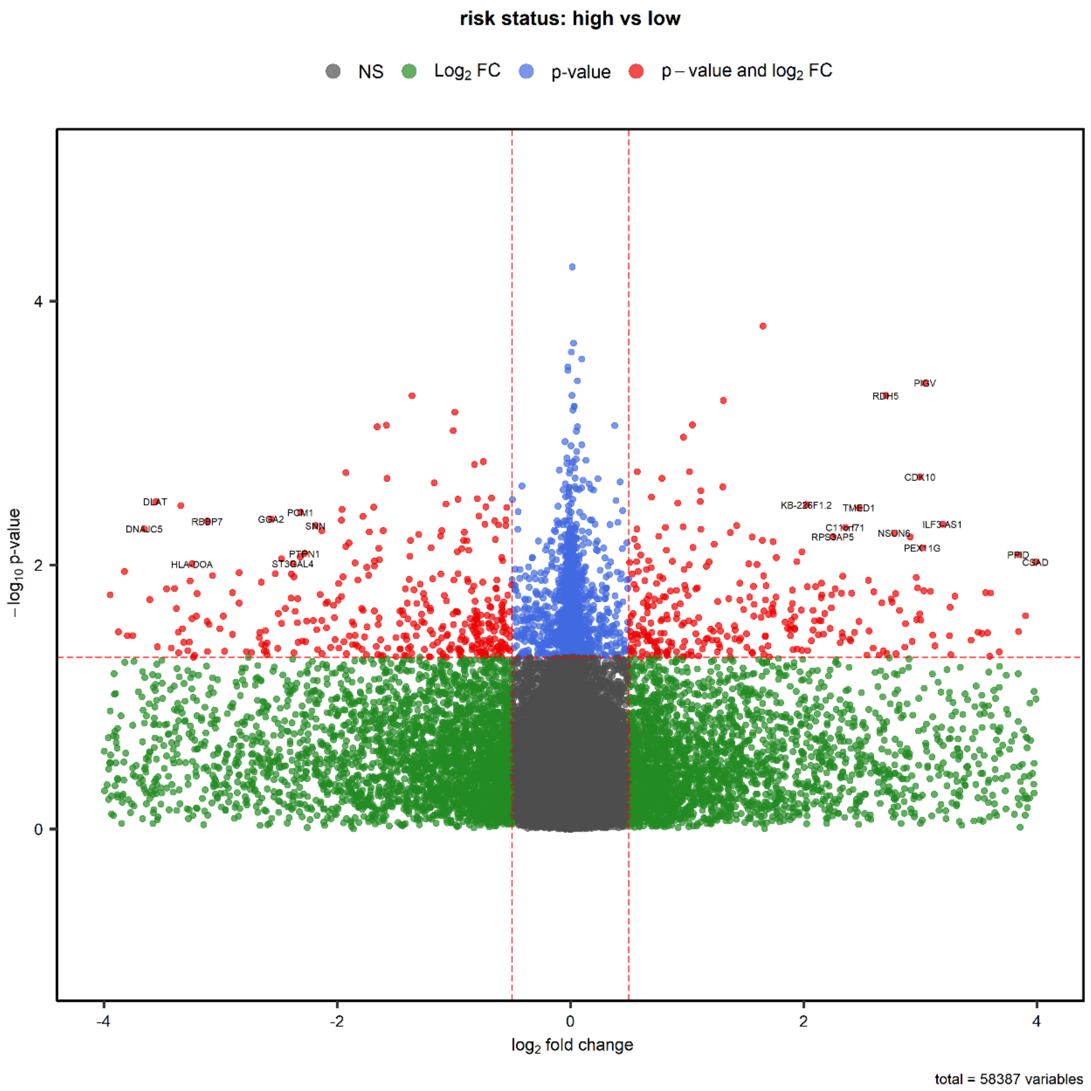

Figure S2 Volcano map of DEGs between high- and low-risk groups

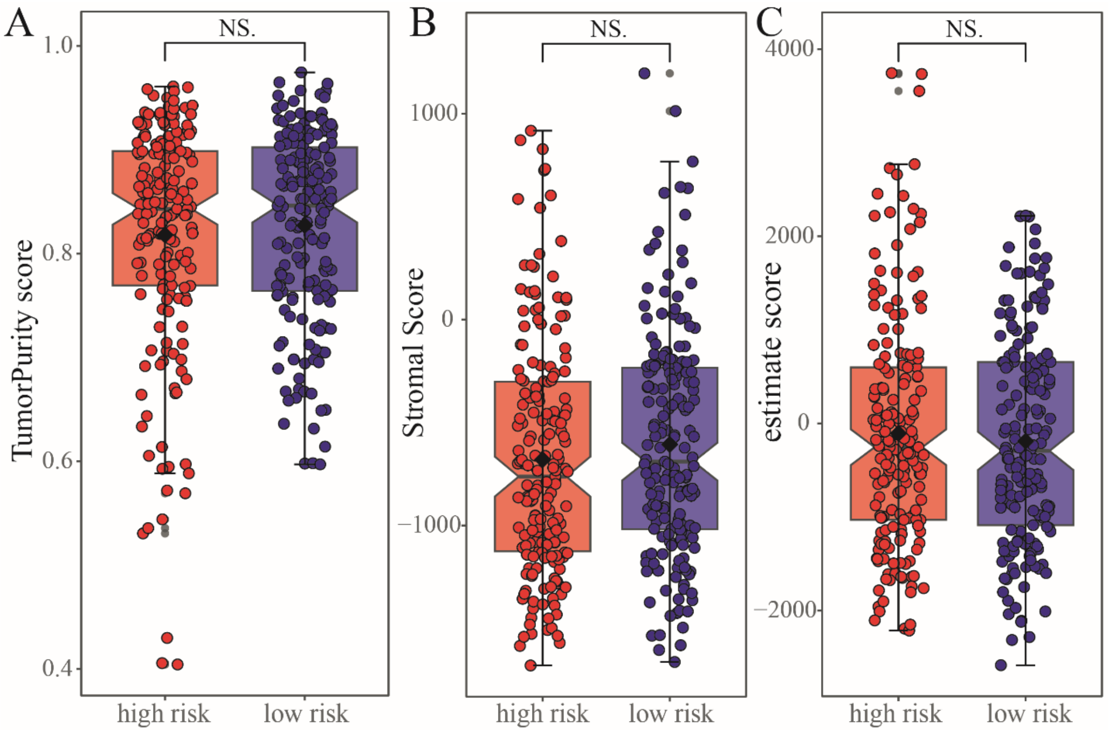

Figure S3 Difference analysis of tumor purity score (A), stromal score (B), and estimate score (C) among high- and low-risk groups based on estimate. (NS means non-significant)

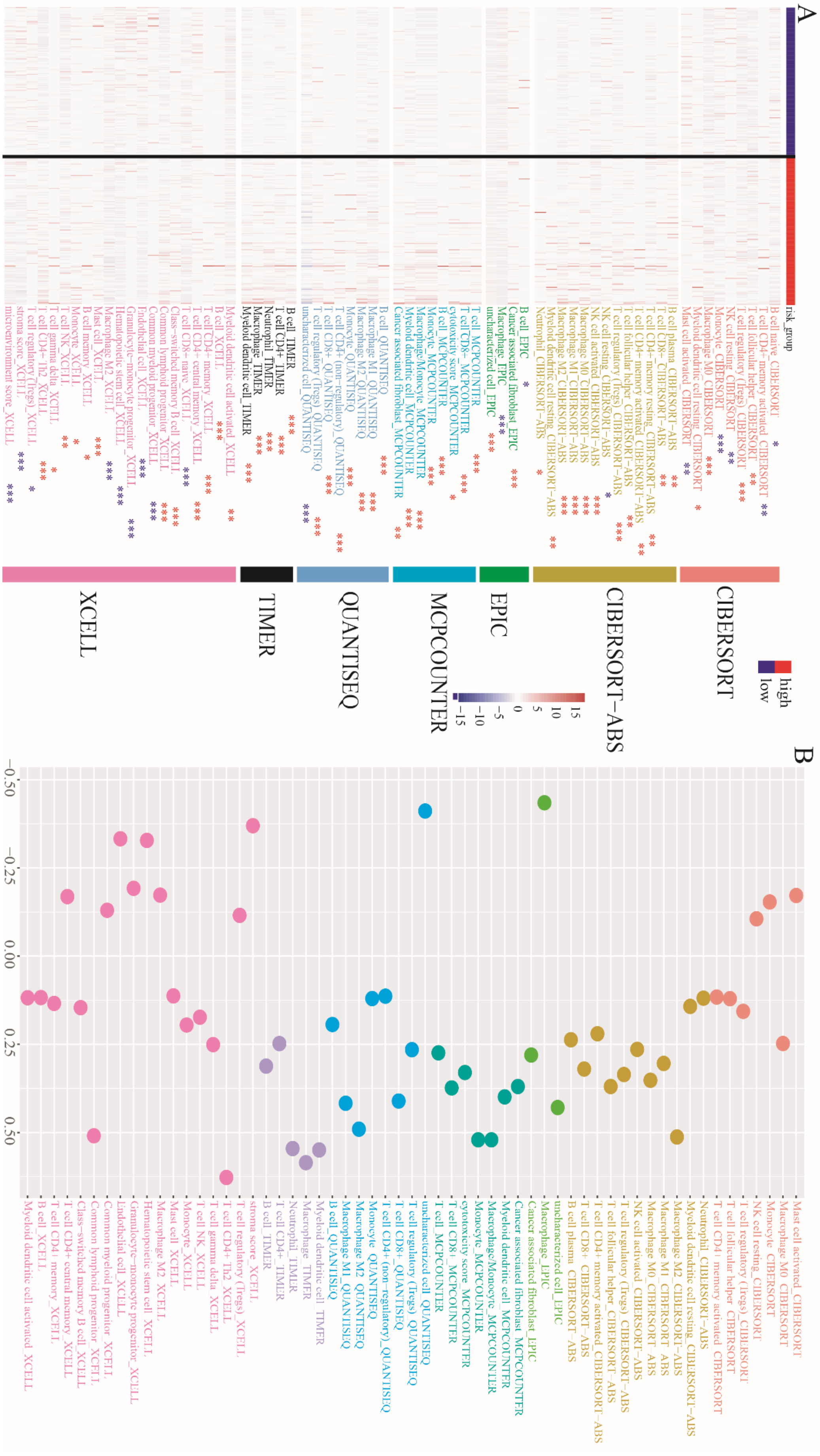
A
B
risk_group
high
low
CIBERSORT
CIBERSORT−ABS
EPIC
MCPCOUNTER
QUANTISEQ
TIMER
XCELL

Figure S4 Immunoinfiltration analysis of liver cancer based on multiple methods. (A) Analysis of the difference of immune infiltration degree between high- and low-risk groups, red asterisks represent significantly high expression in high-group, and blue is the opposite. (B) relationship between immune cell proportion and risk score. (*$p< 0.05$, **$p< 0.01$, ***$p < 0.001$, ****$p < 0.0001$, NS means non-significant)

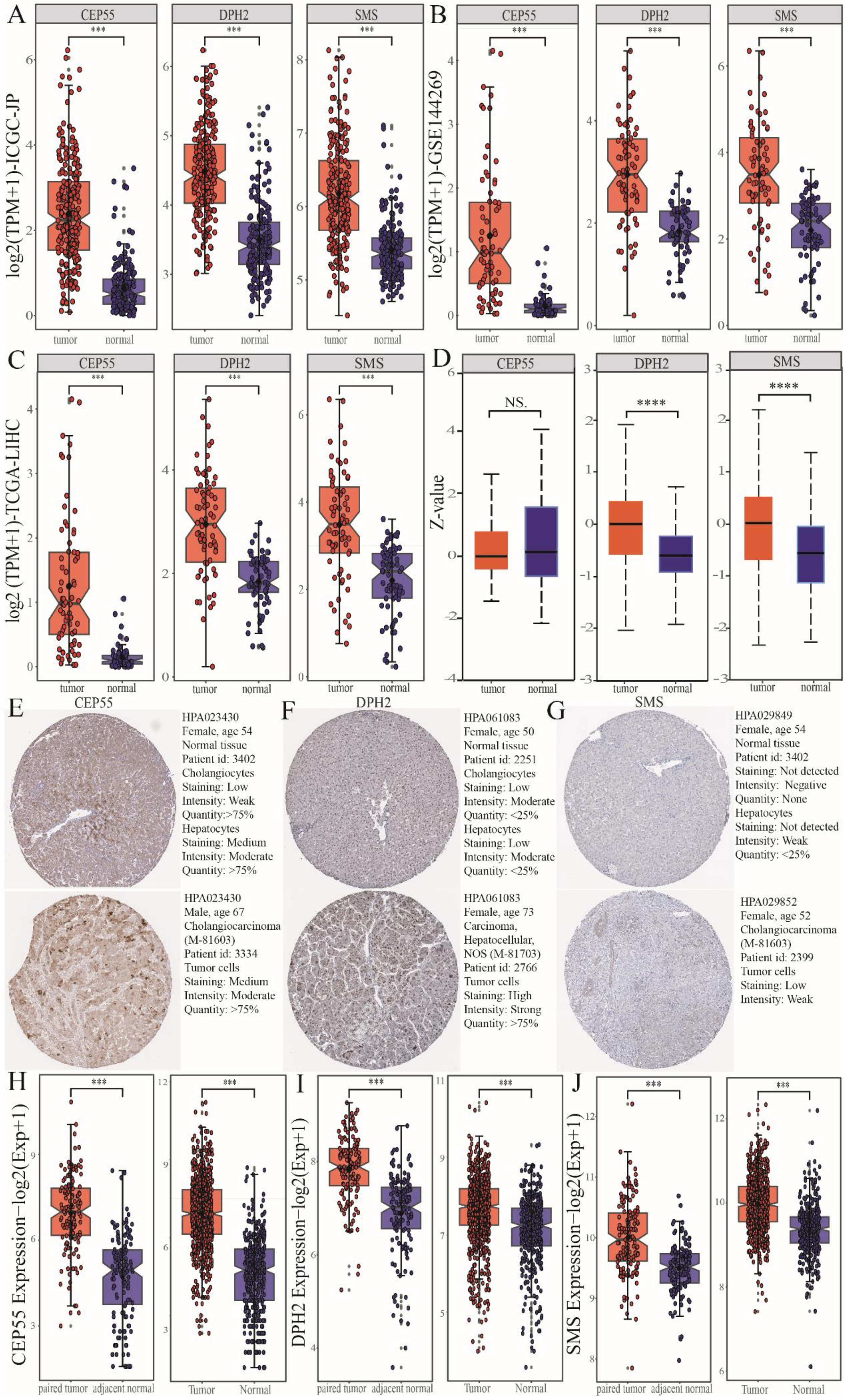
A
B
C
D
E
F
G
H
I
J
CEP55
DPH2
SMS
log2(TPM+1)-ICGC-JP
log2(TPM+1)-GSE144269
log2 (TPM+1)-TCGA-LIHC
Z-value
NS.
****
***
tumor
normal
HPA023430
Female, age 54
Normal tissue
Patient id: 3402
Cholangiocytes
Staining: Low
Intensity: Weak
Quantity:>75%
Hepatocytes
Staining: Medium
Intensity: Moderate
Quantity: >75%
HPA023430
Male, age 67
Cholangiocarcinoma
(M-81603)
Patient id: 3334
Tumor cells
Staining: Medium
Intensity: Moderate
Quantity: >75%
HPA061083
Female, age 50
Normal tissue
Patient id: 2251
Cholangiocytes
Staining: Low
Intensity: Moderate
Quantity: <25%
Hepatocytes
Staining: Low
Intensity: Moderate
Quantity: <25%
HPA061083
Female, age 73
Carcinoma,
Hepatocellular,
NOS (M-81703)
Patient id: 2766
Tumor cells
Staining: High
Intensity: Strong
Quantity: >75%
HPA029849
Female, age 54
Normal tissue
Patient id: 3402
Staining: Not detected
Intensity: Negative
Quantity: None
Hepatocytes
Staining: Not detected
Intensity: Weak
Quantity: <25%
HPA029852
Female, age 52
Cholangiocarcinoma
(M-81603)
Patient id: 2399
Tumor cells
Staining: Low
Intensity: Weak
CEP55 Expression−log2(Exp+1)
DPH2 Expression−log2(Exp+1)
SMS Expression−log2(Exp+1)
paired tumor
adjacent normal
Tumor
Normal

Figure S5 Expression models of risk genes in different databases. Differences in expression of risk genes between tumor and normal samples in ICGC-JP (A) and GSE144269 (B), and TCGA-LIHC (C). (D) Differences in protein expression of risk genes between tumor and normal samples based on UALCAN database. (E) (F) (G) Immunohistochemical analysis of risk genes. (H) (I) (J) Expression difference of risk genes between tumor and normal tissues based on TNMplot. The significance of the difference was analyzed by Wilcoxon signed rank test. (*p< 0.05, **p< 0.01, ***p < 0.001, ****p < 0.0001, NS means non-significant).

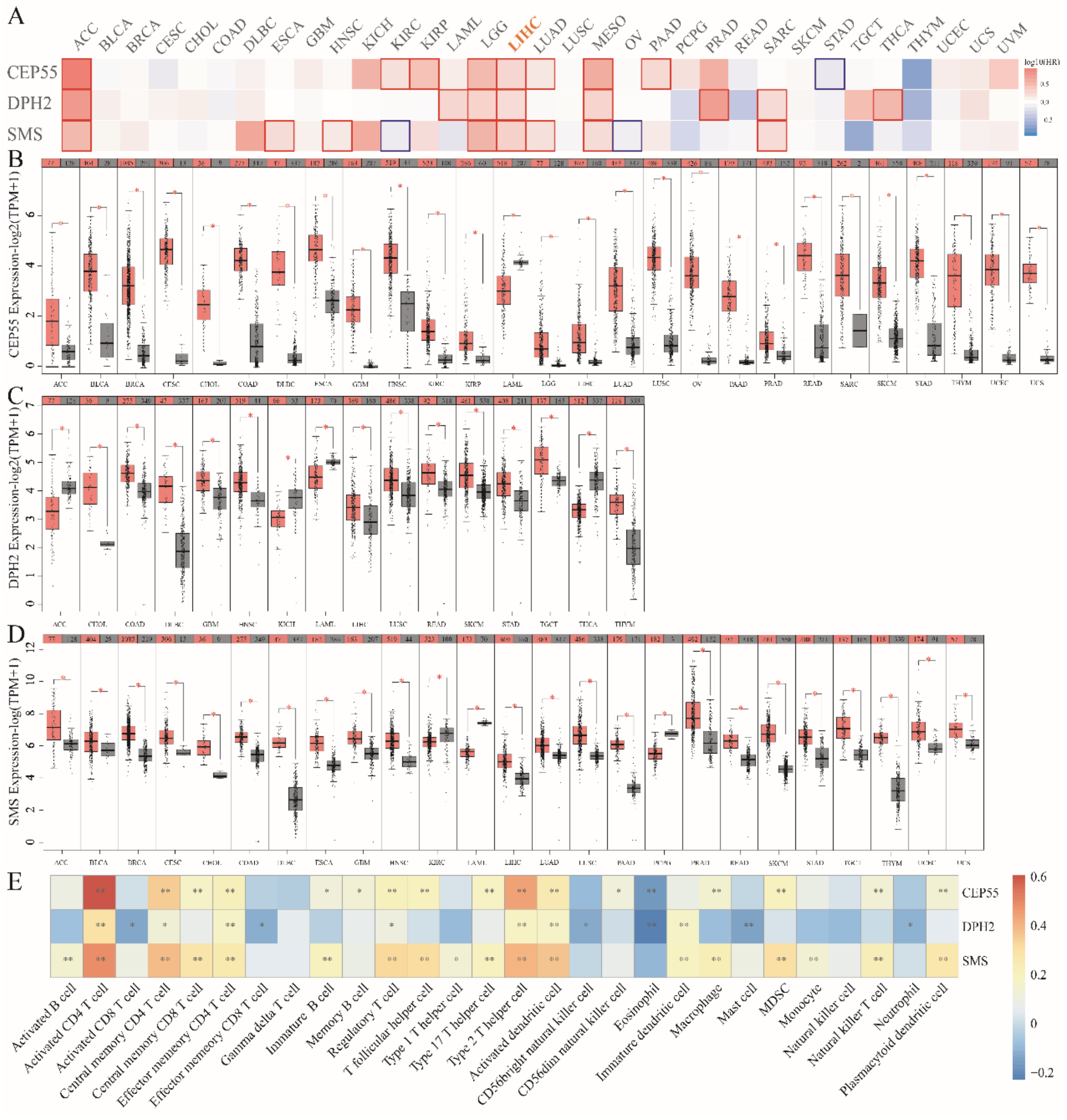

Figure S6 Pan-cancer analysis of risk genes Pan-cancer survival (A) and expression analysis (B) (C) (D). (E) Correlation analysis between risk genes and 28 types of

immune cells. (*p< 0.05, **p< 0.01, ***p < 0.001, ****p < 0.0001, NS means non-significant)

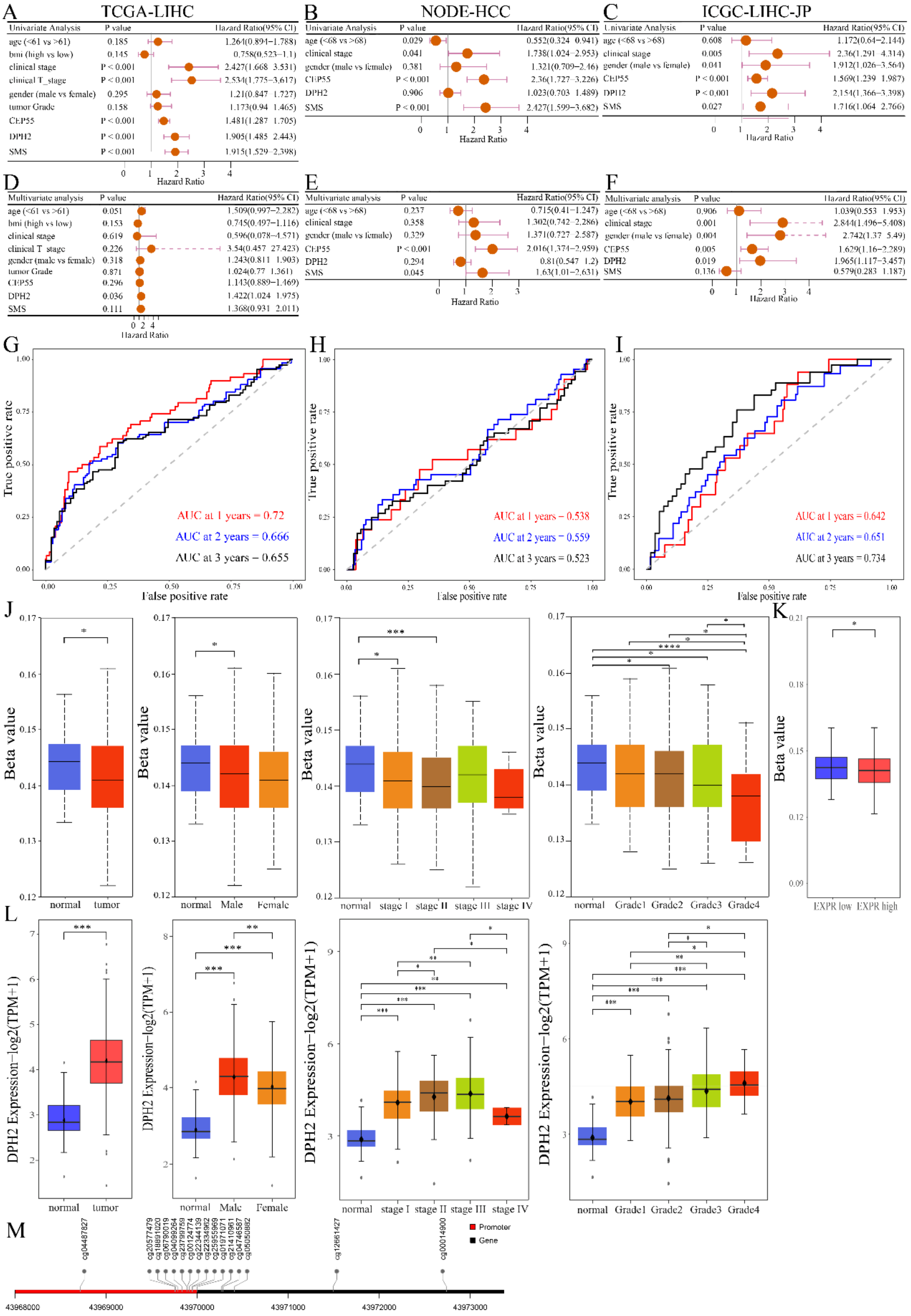

Figure S7 Gene function analysis of DPH2 Independent prognostic analysis of DPH2 for liver cancer based on Univariate Cox regression (A) (B) (C) and Multivariate Cox

regression (D) (E) (F). (G) (H) (I) Prognostic performance of DPH2 at 1, 2, 3 years based on time-dependent ROC analysis. The relationship between DPH2 methylation level (J), gene expression level (L) and clinical indicators. (K) Relationship between methylation levels of DPH2 and its gene expression level. (M) Methylation modification sites of DPH2. The significance of the difference was analyzed by Wilcoxon signed rank test. (A) (D) (G) are the analysis results of TCGA-LIHC, (B) (E) (H) are the analysis results of NODE-HCC, (C) (F) (I) are the analysis results of ICGC-LIIHC-JP. (*p< 0.05, **p< 0.01, ***p < 0.001, ****p < 0.0001, NS means non-significant)

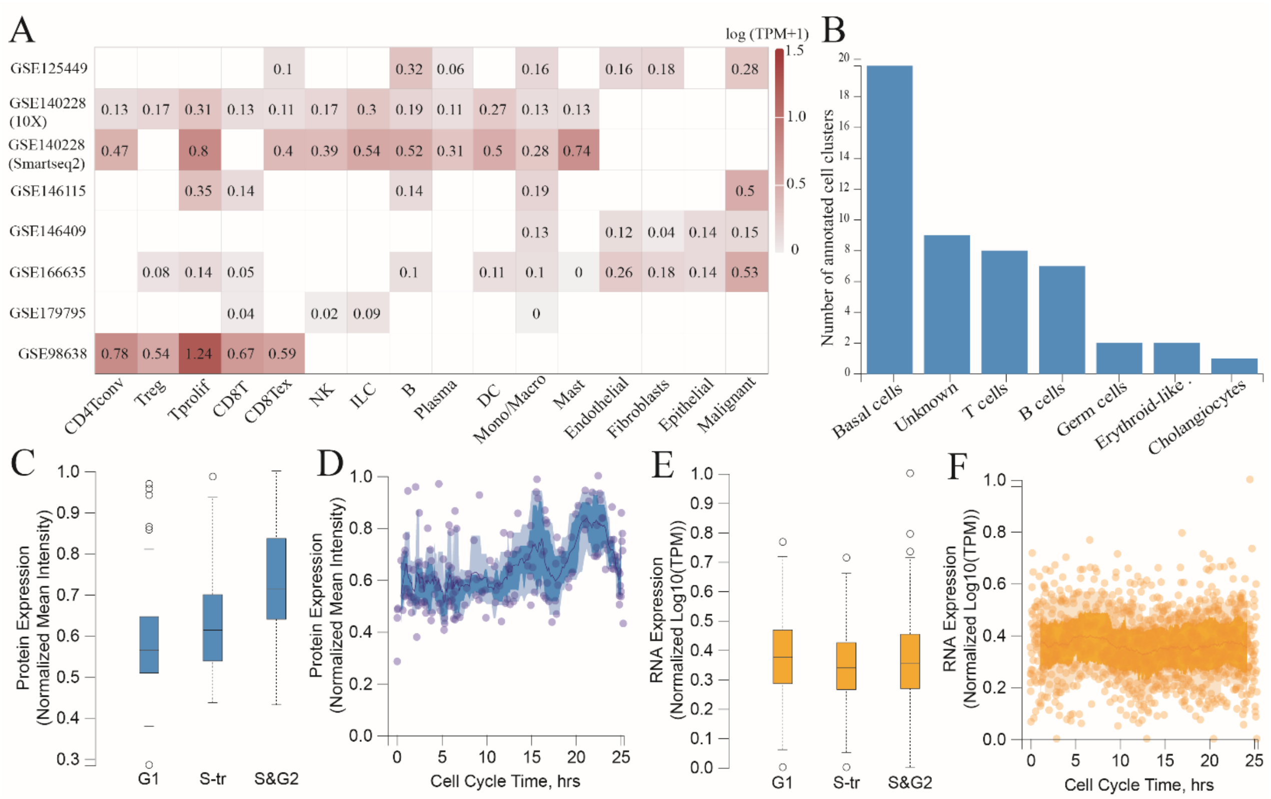

Figure S8 Single cell expression of DPH2 (A) The expression levels of risk genes across different cell types in multiple liver cancer single-cell datasets. (B) Cell marker analysis of DPH2. The relationship between the expression levels of DPH2 at both protein (C) (D) and transcript levels and the cell cycle (E) (F).
